# Poly(butylene succinate) Film Coated with Hydroxypropyl Methylcellulose with Sea Buckthorn Extract and Its Ethosomes—Examination of Physicochemical and Antimicrobial Properties Before and After Accelerated UV Aging

**DOI:** 10.3390/polym17131784

**Published:** 2025-06-27

**Authors:** Szymon Macieja, Magdalena Zdanowicz, Małgorzata Mizielińska, Wojciech Jankowski, Artur Bartkowiak

**Affiliations:** Center of Bioimmobilisation and Innovative Packaging Materials, Faculty of Food Sciences and Fisheries, West Pomeranian University of Technology in Szczecin, Janickiego St. 35, 71-270 Szczecin, Poland; szymon.macieja@zut.edu.pl (S.M.); mzdanowicz@zut.edu.pl (M.Z.); mmizielinska@zut.edu.pl (M.M.); jw42757@zut.edu.pl (W.J.)

**Keywords:** accelerated aging, active packaging, ethosomes, poly(butylene succinate), hydroxypropyl methylcellulose, sea buckthorn extract

## Abstract

The new generation of food packaging should not only be biodegradable, but also provide additional protective properties for packaged products, extending their shelf life. In this paper, we present the results of research on cast-extruded poly(butylene succinate) (PBS) films coated with hydroxypropyl methylcellulose (HPMC) modified with CO_2_ extract from sea buckthorn (ES) or its ethosomes (ET) at amounts of 1 or 5 pph per HPMC. In addition, the developed films were exposed to accelerated aging (UV radiation and elevated temperature) to determine its effect on the films’ properties. Based on SEM, it can be concluded that accelerated aging results in the uncovering of the extract and ethosomes from the coating’s bulk. GPC showed a decrease in the molecular weight of PBS after treatment, additionally amplified by the presence of HPMC. However, the addition of ES or ET in low concentrations reduced the level of polyester degradation. The presence of the modified coating and its treatment increased the oxygen barrier (a decrease from 324 cm^3^/m^2^ × 24 h for neat PBS to 208 cm^3^/m^2^ × 24 h for the coated and modified PBS ET5). Despite the presence of colored extract or ethosomes in the coating, the color differences compared with neat PBS were imperceptible (ΔE < 1). The addition of 5 pph of sea buckthorn extract or its ethosomes in combination with accelerated aging resulted in the complete inhibition of the growth of *E. coli* and *S. aureus*, which was not observed in non-aged samples. The results obtained demonstrate an improvement in bioactive properties and protection against the negative effects of UV radiation on the film due to the presence of ET or ES in the coating. The developed systems could be used in the food industry as active packaging.

## 1. Introduction

Packaging made of polyethylene (PE), polypropylene (PP) or poly(ethylene terephthalate) (PET) dominates the market due to its low price, good barrier properties (better barrier properties towards polyolefins or polylactide), and mechanical/thermal resistance [1]. However, its persistence in the natural environment (decomposition time as long as hundreds of years) leads to the accumulation of microplastics in ecosystems [2]. Eight million tons of microplastics enter the oceans annually [3], with global plastic production exceeding 460 million tons [4] and only a 10% recycling rate [5]. According to the 2023 United Nations Environment Programme report, the annual global cost of plastic pollution—including social and environmental costs—is a truly overwhelming figure, estimated to be between USD 300 and 600 billion [6].

Promising alternatives for petroleum-based polymers include biopolymers such as polylactide (PLA), polyhydroxyalkanoates (PHA), and bio-based poly(butylene succinate) (PBS). PBS, an aliphatic polyester synthesized from petrochemical feedstocks or biomass (e.g., by biotechnological synthesis of succinate) [7,8,9], is distinguished by its thermoplasticity (melting point: ~114 °C), crystallinity (~40–60%) [10], and high degree of biodegradability [11].

To meet these conditions, the packaging must either act as a barrier to oxygen (allowing food to be packaged under MAP or vacuum conditions) or be effective/active against microorganisms responsible for food spoilage [12,13]. Unfortunately, PBS is not a high-barrier material, nor does it exhibit antibacterial properties [14,15]. Covering PBS with a coating containing substances that exhibit antimicrobial properties and simultaneously decrease oxygen transmission can be a solution.

Hydroxypropyl methylcellulose (HPMC) is a cellulose derivative that is water soluble and capable of forming transparent, flexible films. It is characterized by good compatibility with other biopolymers and active substances, as well as by non-toxicity. Moreover, HPMC has natural barrier properties against oils and dusts, and its aqueous solutions allow essential oils, plant extracts, and nanoparticles to be incorporated into its matrix [16].

Incorporating essential oils and plant-based extracts into packaging systems improves their antimicrobial and antioxidant properties. However, due to their high volatility, low thermal stability, and strong aroma, it is often necessary to encapsulate them in carriers such as liposomes, ethosomes, or nanoemulsions. The literature indicates that the use of such carrier systems allows the longer release of active ingredients, reduces their loss during heat treatment, and increases their effectiveness [17].

Sea buckthorn (*Hippophae rhamnoides*) extract is a rich source of bioactive compounds such as tocopherols, carotenoids, phytosterols, and unsaturated fatty acids [18]. Supercritical CO_2_ extraction serves as a highly effective method for extracting lipophilic bioactive compounds, such as carotenoids, tocopherols, and unsaturated fatty acids. By regulating temperature and pressure, CO_2_ ensures the creation of pure, biologically potent extracts while safeguarding heat-sensitive components from thermal degradation [19]. Studies on PBS film modified with scCO_2_ extract have shown its strong antioxidant and antimicrobial activity against Gram-positive and Gram-negative bacteria, such as *Staphylococcus aureus* and *Escherichia coli* [20]. Thanks to these properties, the oil has known applications in cosmetics and as a natural preservative in food products. Its use in active packaging films can significantly extend the shelf life of food [21].

Ethosomes are phospholipid structures similar to liposomes, containing high concentrations of ethanol (10–45% by volume), which increases membrane fluidity and allows better penetration through hydrophilic and lipophilic layers [22]. In food technology, including packaging, they can act as carriers of active (i.e., antimicrobial, antioxidative) ingredients, controlling their release and stabilizing thermolabile, active ingredients such as essential oils and extracts. Unlike classical liposomes, they are stable at pH 4–9 [23].

The aim of the presented study is to modify biodegradable polyester packaging material with functionalized coating based on polysaccharide derivative to obtain fully bio-based and environmentally friendly (including basing material, coating, and additives) material that can be used, e.g., for food packaging or medical packaging. Hydroxypropyl methylcellulose was used as a coating carrier of the additives as a greener alternative for non-biodegradable synthetic commercial coating carriers. A coating carrier was functionalized with scCO_2_ sea buckthorn extract and their ethosomes. This type of material modification was used to avoid the overheating of theactive agent (as in the case of the introduction into polymer matrix during regranulation). The physicochemical (mechanical properties, barrier towards oxygen, morphology studied by FTIR and SEM, average molecular weight, and contact angle of the surface) and antimicrobial properties of PBS films before and after accelerated aging (UV and raised temperature) were evaluated. Such accelerated treatment was performed in order to investigate how this packaging might react to long-term storage in outdoor conditions.

## 2. Materials and Methods

### 2.1. Materials and Reagents

Poly(butylene succinate) (FZ91PM BioPBS^TM^) was purchased from (BioPBS, PTT MCC Biochem Co. Ltd., Rayong, Thailand).

Hydroxypropyl methyl cellulose (HPMC) was purchased from Wolff Cellulosics GmbH & Co. KG (Walsrode, Germany). Decyl glucoside and caprylyl/capryl glucoside were provided by Zielonyklub (Kielce, Poland). Subcritical CO_2_ sea buckthorn extract (scCO_2_) was purchased from ECOSPA (Warszawa, Poland). Agar-agar, plate count agar, and potato dextrose agar were purchased from Merck (Darmstadt, Germany); ethanol 96% from POCH (Poland); and lecithin with 50% content of phosphatidylcholine from LecFis PC 50 IPM, Fismer (Hamburg, Germany). All the chemicals were of analytical grade. *Escherichia coli* DSMZ 498, *Staphylococcus aureus* DSMZ 346, and *Candida albicans* ATCC 10231 were procured from ATCC (American Type Culture Collection, Manassas, VA, USA).

The ethosomes containing scCO_2_ sea buckthorn extract in forms of aqueous dispersion were provided by the Bartko Consulting company (Szczecin, Poland). The ethosomes were obtained using the modified classic method [24]. The final concentration of scCO_2_ sea buckthorn extract/lecithin-PC50/96%ethanol in final ethosome dispersions was 1/1/10 wt. % in distilled water.

### 2.2. Preparation of PBS Films via Cast Extrusion

PBS foil was obtained by cast extrusion. The dry PBS pellets (at 70 °C, 3 h) were extruded through a flat die using a Chill-Roll Cast Film Extrusion Line, Type LCR-300 Co-Ex; L/D 30:1, with a screw diameter of 20 mm and three heating zones (Labtech Engineering, Samut Prakan, Thailand), to obtain films with a thickness of 75 to 90 µm. The average temperature profiles during the tests were 160 °C. PBS foil was stored in ambient conditions (23 °C, RH 50%).

### 2.3. Preparation of Coated Films

An aqueous HPMC solution (2 wt. %) was prepared by adding polymer into distilled water and stirring using a magnetic stirrer at room temperature, and decyl glucoside and caprylyl/capryl glucoside were introduced at a rate of 0.1 g per 1 g of HPMC as plasticizers. Then, subcritical CO_2_ sea buckthorn extract (ES) or its ethosomes ES were added to the polymer compositions to obtain 1 or 5 wt parts per 100 wt parts of HPMC in the final coating.

A coating layer on PBS films was obtained using a Unicoater 409 coating machine (Erichsen, Hemer, Germany) equipped with a 20 µm diameter roller, moving at a speed of 50 mm/s. A volume of 1.5 mL of the film-forming solution was applied to PBS and evenly spread using the roller. The solvent was evaporated in a dryer with a fan at 60 °C for 20 min. The films’ acronyms are shown in Table 1.

### 2.4. Accelerated UV Aging of the Films

Neat PBS, HPMC-coated films were all cut into rectangles (235 × 70 mm). The aforementioned samples were introduced into a weathering chamber (Q-LAB DEUTSCHLAND GMBH, Saarbrucken, Germany) with the following settings: wavelength of 340 nm (UV-A), 0.60 W/m^2^, RH 60% irradiated for 72 h at 60 °C.

### 2.5. Thickness and Mechanical Properties

A digital micrometer (Dial Thickness Gauge 7301, Mitutoyo Corporation, Kanagawa, Japan, accuracy of 0.001 mm) was used to measure film thickness at ten random points on each sample, with results expressed as mean ± standard deviation.

A mechanical test was performed using a Zwick/Roell Z2.5 tensile tester (Ulm, Germany), as described elsewhere [25]. Briefly, films were cut into 10 mm wide strips with an initial grip separation of 50 mm and a crosshead speed of 10 mm/min. A minimum of five replicate samples were analyzed. TestXpert III (v1.61) software was used to calculate the elongation at break (EB) and maximum tensile strength (TS) with standard deviations. Puncture was performed according to the method described in the work [26].

### 2.6. Spectral Analysis of the Films

Transmission infrared spectrometry with Attenuance Total Reflectance (FTIR-ATR) of the films was performed at room temperature using a Perkin Elmer Spectrum 100 FTIR spectrometer (Waltham, MA, USA). Measurements were taken in the range of 4000–650 cm^−1^ with 16 scans at a resolution of 4 cm^−1^. SPECTRUM software (version 10.03.06.0100) was used to record the spectra and their baseline correction.

### 2.7. SEC of Neat PBS and the Samples After Accelerated UV Aging

The molecular weight of non-aged and UV-aged PBS films was determined by high-performance size exclusion chromatography (HPSEC) using an S1000 pump, an S2300 refractive index detector, and a 100 µL sample loop (Knauer, Berlin, Germany). Prior to analysis, the HPMC coating from ET or ES was washed off using distilled water in order to test the PBS films alone. Separation was performed using a PFG, Linear-XL, 8 × 300 mm, 7 µm column (Agilent Technologies, Santa Clara, CA, USA), at room temperature and a flow rate of 1.5 mL min^−1^. The eluent composition was chloroform (p.a.). Polystyrene standards were used for the calibration and determination of the relative molecular weight.

### 2.8. Films Color Analysis

Color measurements were conducted using a CR-5 colorimeter (Konica Minolta, Tokyo, Japan) employing the CIELab color scale. For each of the films examined, ten measurements were taken at some random points. The total color difference (ΔE) and yellowness index (YI) were determined according to the method presented in the previous work [13].

The opacity was determined using an Opacimeter EE Model 12 (Diffusion Systems Ltd., London, UK). The opacimeter was pre-calibrated using a standard white plate (value 100 ± 1, Diffusion Systems Ltd., London, UK), and measurements were taken from each film on ten occasions and presented as mean ± standard deviation.

The transparency of the films was measured using a Thermo Scientific (Waltham, MA, USA) Evolution 220 UV–VIS spectrophotometer. Strips of films, with a surface area matching the quartz cuvette (5.5 cm × 1 cm), were placed along with a cuvette in the apparatus, and transmittance at 700 nm was recorded.

### 2.9. Oxygen Permeability Analysis

The oxygen transmission rate (OTR) for the PBS foil and coated films was determined using the OX-TRAN Model 2/10 (Mocon, Minneapolis, MN, USA) in accordance with ASTM D3985 [27]. The area of the analyzed sample was 5 cm^2^. The tests were performed at a temperature of 23 °C and a relative humidity (RH) of 50%.

### 2.10. Contact Angle Measurement

The contact angle determination of the films’ surface was performed using an SEO contact analyzer, Phoenix-Mini (PM-041807, Suwon, Republic of Korea). Surface contact angle values were calculated using Surfaceware 8 software after distilled water drop deposition.

### 2.11. Antimicrobial Analysis

Antimicrobial activity tests were conducted following a modified version of ASTM E 2180-01, as detailed in [20]. Briefly, film samples (30 mm × 30 mm) were cut and sterilized using UV light. Agar slurries were created by mixing 0.15 g agar-agar and 0.45 g NaCl in 50 mL of distilled water, and then sterilized. Once cooled, these slurries were combined with 1 mL of microbial suspensions at a concentration equivalent to 0.5 on the McFarland scale. The resulting mixtures (1 mL) were then carefully applied to the sample surfaces and incubated for 24 h at 37 °C with 90% relative humidity. Following incubation, the samples were carefully removed from the Petri dishes, placed in 100 mL of sterile 0.9% NaCl solution, and thoroughly vortexed. Serial dilutions were prepared, and cultures were grown on plate count agar (for *E. coli* and *S. aureus*) and potato dextrose agar (for *C. albicans*). These cultures were then incubated at 37 °C for 24 h. The findings are presented as mean values with standard deviations.

### 2.12. Scanning Electron Microscopy Evaluation

The surface of the samples was examined using SEM (scanning electron microscope). Preliminarily, the samples were placed on pin stubs and covered with a thin layer of gold in a sputter coater at room temperature (Quorum Technologies Q150R S, Laughton, East Sussex, UK). Next, SEM micrographs were obtained using a Vega 3 LMU microscope (Tescan, Brno-Kohoutovice, Czech Republic). A microscopic examination was performed using a tungsten filament with an accelerating voltage of 10 kV.

### 2.13. Statistical Analysis

Statistical analysis was performed using Statistica version 13 software (StatSoft Poland, Krakow, Poland). Differences between means were determined using an analysis of variance (ANOVA), followed by Fisher’s post hoc LSD test at a significance threshold of *p* < 0.05.

## 3. Results and Discussion

### 3.1. SEM

**Figure 1:**
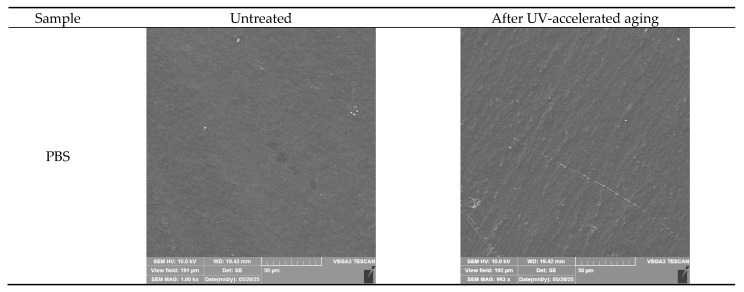

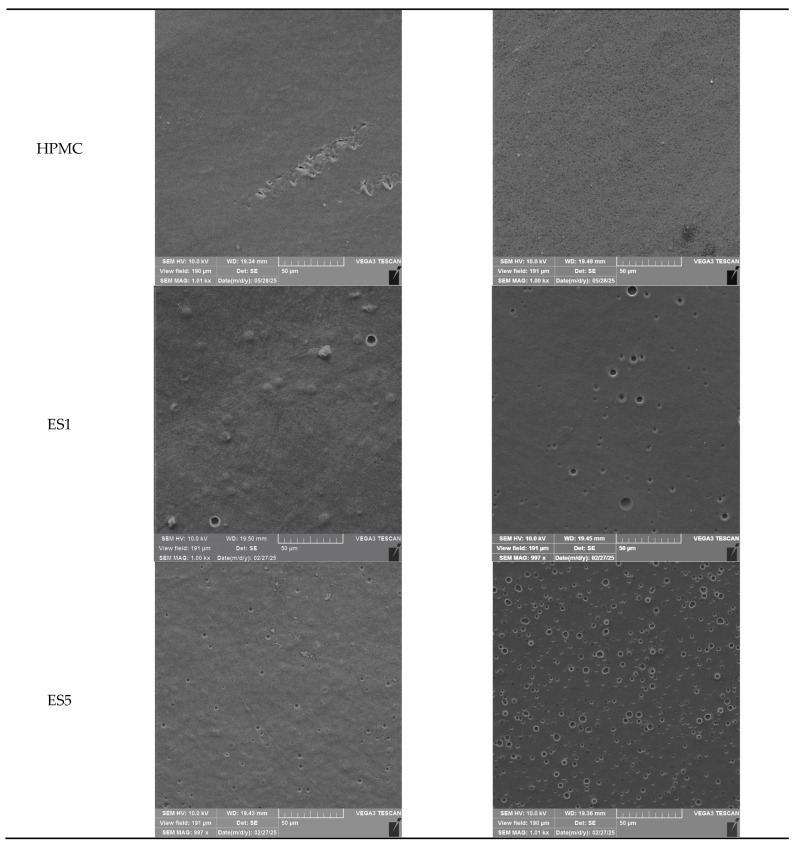

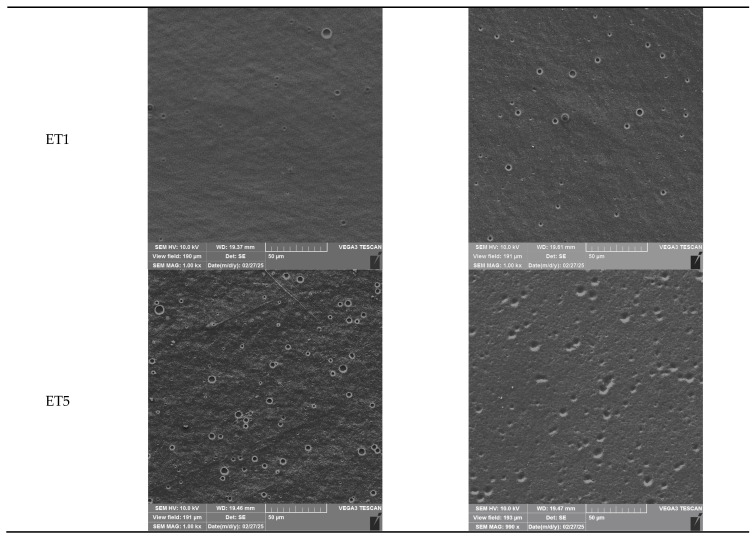
The samples’ surface before and after UV aging.

Figure 1 shows scanning electron microscope images of the films and coatings’ surface before and after accelerated UV aging. The neat PBS film had a smooth, homogeneous surface. As a result of UV exposure, slight wrinkles became visible on the surface. A similar but stronger effect was observed in the work of Scolaro et al., in which PBS (the same type of PBS from the same supplier) was artificially aged using UVC radiation. As a result, the surface of the film changed, forming reliefs and filaments [28].

HPMC layer on PBS also appeared uniform and homogeneous, with some minor imperfections. However, after aging, changes in the microstructure were noticeable. This may be related to the changes in the crystallinity of HPMC films under the influence of UV radiation observed by Rao et al. [29]. Their XRD analyses show that microcrystalline parameters (crystallite size (LXRD) and crystallinity (Xc)) are decreasing with UV irradiation due to the photo-degradation process.

**Figure 2:**
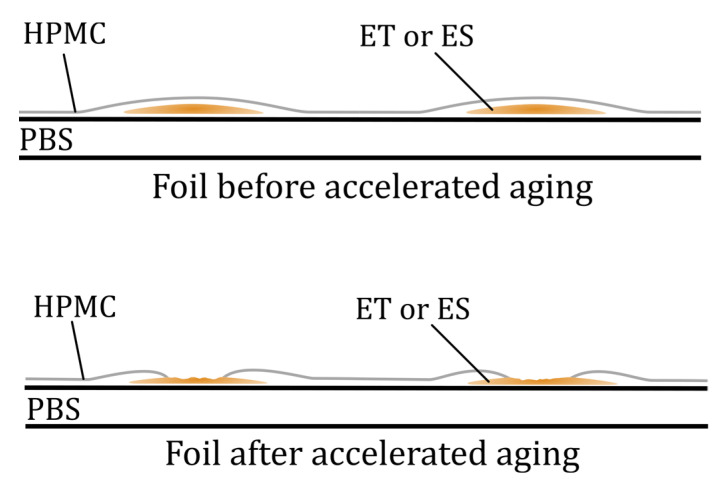
Scheme of coated PBS modification during/after accelerated aging.

The film-forming mixtures formed emulsions after the introduction of the additives, and then, after application onto the PBS film, caused the encapsulation and “covering” of ES and ET particles enclosed within the coating reflected as some bumps and individual spots visible in PBS ES1 and PBS ET micrographs. In the case of PBS ES5 and PBS ET5 samples, there were more spots where the inclusions were present on the surface, which was obviously due to the higher proportion of the additives in the coating bulk. As a result of accelerated aging, the additives’ particles migrated to the surface, forming larger amounts of the spots in the top layer. Based on SEM micrographs’ analysis, it can be assumed that accelerated aging caused the erosion of HPMC layer, thereby exposing ES or ET encapsulated in the coating, without total release from the material. The mechanism of the changes is proposed in Figure 2. This assumption was confirmed by further microbial study and the high antimicrobial activity of the aged coatings (see Section 3.7). If the substances had been released from the material during/after treatment, the coatings would no longer exhibit antimicrobial activity. This result indicates the controlled migration of the ES or ET from the bulk of the coating layer to its surface and their exposure to conditions of aging, which may have a beneficial effect on improving the functional properties of the films, making them more active over time. Contrary results were observed in our previous work [13], which showed that small scratches were visible on the surface of the irradiated and nonirradiated active coatings, confirming that accelerated aging had no impact on the surface of a polyester blend film covered with the extract of *Hippophae rhamnoides* L., *Hypericum* L., and *Achillea millefolium* L. That could be related to the hydrophilic nature of herbal extracts’ mixture that was miscible with HPMC.

### 3.2. Contact Angle of PBS Surface

Table 2 lists the contact angle values for native PBS and modified films before and after accelerated aging. The highest parameter’s value (63°) was obtained for unmodified PBS, and the lowest for PBS coated with HPMC (32°). The drop of the contact angle value for treated PBS can be caused by changes in the morphology. SEM (Figure 1) evidenced that its surface is less smooth than that of native PBS. Polysaccharide-modified PBS exhibited the highest wettability due to the highly hydrophilic nature of HPMC (resulting from, e.g., OH groups and dissolution in water) and the presence of the plasticizer that decreased surface tension (confirmed by SEM, Figure 1). The increase in contact angle value after UV treatment can be caused by the erosion of the external layer, revealing less hydrophilic PBS. It can be noticed that the addition of ES and ET into HPMC decreases wettability (the higher content of the additives, the higher the contact angle values) due to the hydrophobic character of ES and ET. The decrease in the values of the parameter after accelerating aging can be caused by surface alternation after exposition to the irradiation and partial release of the additives (Figure 2).

### 3.3. Spectral Analysis of the Films

**Figure 3:**
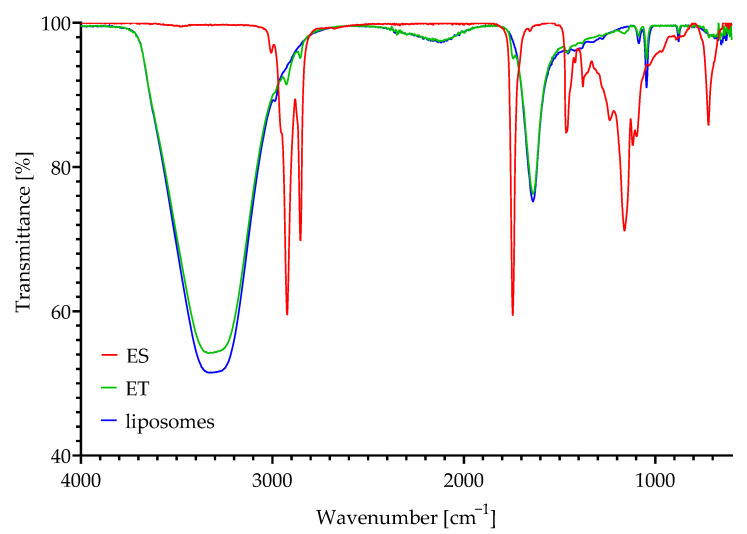
The FTIR spectra of scCO_2_ sea buckthorn extract (ES), scCO_2_ sea buckthorn extract-based ethosomes (ET), and liposomes acting as the carrier in the ET system.

Figure 3 shows the FTIR spectra of ES, liposomes/phospholipids as a carrier for the extract in ethosomes, and ET.

The ES spectrum shows peaks with high intensity at 2856 cm^−1^ and 2925 cm^−1^, which are assigned to the aliphatic CH_2_ symmetric and asymmetric stretching vibration, respectively. At 1754 cm^−1^, a high-intensity peak associated with carbonyl group vibration can be observed. The bands at 1180–1076 cm^−1^ are associated with the vibration of C–O ester groups and CH_2_ groups. The obtained spectrum coincides with literature data for sea buckthorn extracts obtained using supercritical CO_2_ [30]. Spectra for phospholipid-based composition and for ET show three main bands. The broad band at 3750–2850 cm^−1^ is associated with OH groups from ethanol stretching, while peaks at 1634 cm^−1^ and 1052 cm^−1^ are associated with C=O bond stretching and C–O bond presence, respectively. These results correspond to data for ethosomes presented in the literature [31]. The spectra obtained for the carrier and ET are quite similar, but the small peaks characteristic of ES described above are observable for ET. This indicates that although most of the signal comes from the carrier with ethanol, the low-intensity signal from the ES encapsulated/scattered in the carrier is also visible.

**Figure 4:**
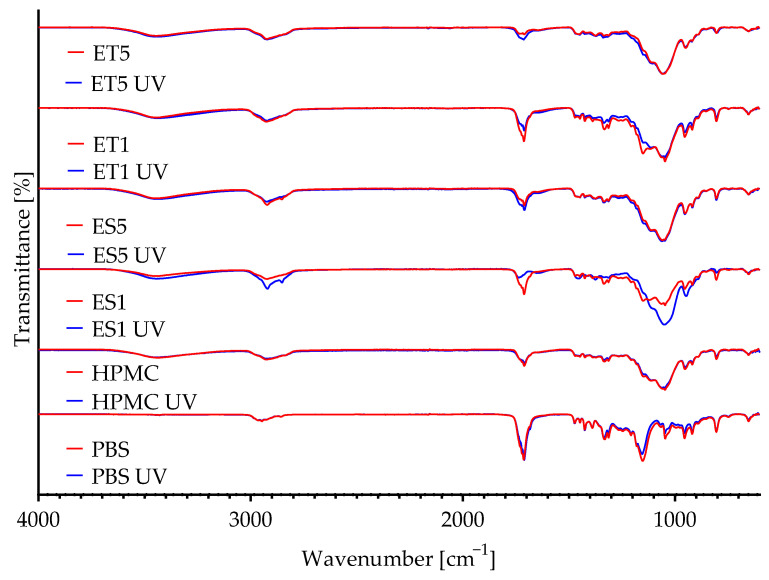
The FTIR spectra of PBS films, PBS films coated with HPMC, and PBS films coated with HPMC containing sea buckthorn scCO_2_ extract or ethosomes before and after UV aging.

The FTIR spectra of PBS, PBS/HPMC, and PBS/HPMC samples with ES or ET, both before and after UV exposure, are shown in Figure 4. A strong band at 1719 cm^−1^ was observed in all samples, attributed to the stretching of ester groups (C=O) characteristic of PBS. In addition, bands resulting from the C–O group at 1149 cm^−1^ and the C–H group at 2949 cm^−1^ were noticed. These bands are characteristic of PBS [13], and their presence in coated samples may indicate the presence of a thin coating layer and signal penetration. In HPMC-coated samples, a slight band at 3423 cm^−1^ assigned to axial stretching OH groups can be seen. It is worth noting that the same band was also observed for the ethosomes’ carrier. HPMC-containing coatings showed more pronounced bands in the 1041 cm^−1^ region, corresponding to C–O–C vibrations in cellulose ether groups [32]. Bands in the range of 2927 and 2849 cm^−1^ (C–H stretching) were present in all modified samples, confirming the participation of ES and ET in the coating structure.

### 3.4. Study of Influence of UV Aging and Type of Coating on the Average Molecular Weight—GPC Results

Table 3 shows the GPC results for an unmodified film and PBS modified with different coatings before and after aging. It can be seen that intensive exposure to UV radiation and elevated temperature during aging caused a decrease in Mn and Mw values and an increase in PDI values. The highest decrease in molecular weight of the polyester was obtained for the film coated with pure HPMC. This may be caused by their hydrophilic character and OH groups facilitating PBS degradation. The incorporation of ES or ET into the coating led to a lower drop of Mn and Mw (in comparison with pure HPMC). However, lower amounts of the additives resulted in a more pronounced protective effect.

### 3.5. Mechanical Properties

PBS foil thickness was in the range of 74–85 µm (it was not possible to determine film grammage due to the differences in the unmodified thickness). The mechanical test results presented in Table 4 show that poly(butylene succinate) became much more brittle after accelerated aging (TS was almost 40% lower; EB dropped from ca. 276% to 6.4%), which is related to the partial degradation of polymer chains during treatment. The negative effect of accelerated aging on the mechanical properties is slightly restricted by the presence of HPMC or the layer with higher content of ES and ET. Interestingly, the best shielding effect (EB dropped to 71%, higher puncture force and penetration path after irradiation) was obtained for ET5, even a quite big difference in Mw in comparison with unmodified PBS. This phenomenon can be a result of the migration of ES and phospholipids from HPMC matrix facilitated by the conditions of accelerated aging towards hydrophobic (more compatible with ES and ET) PBS and act as a plasticizer.

### 3.6. Barrier Properties Towards Oxygen

**Figure 5:**
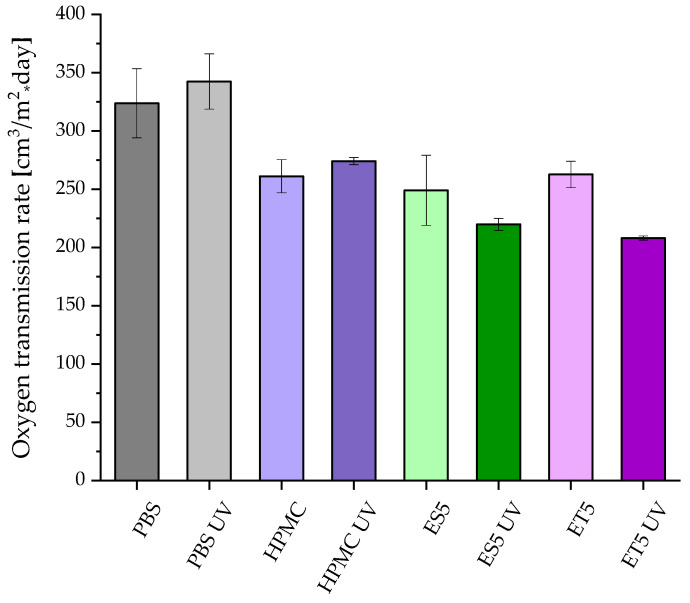
Oxygen transmission rate (OTR) at RH 50% for PBS and coated PBS films before and after UV exposure.

The oxygen transmission rate (OTR) was determined for unmodified PBS, PBS coated with HPMC, and coatings with higher content of ES and ET before and after accelerated aging (Figure 5). The OTR for native PBS film was 324 cm^3^/m^2^ × 24 h (230 cm^3^/m^2^ × 24 h at 0% RH) and slightly increased to 342 cm^3^/m^2^ × 24 h. This can be related to the decrease in the Mw (see Table 2) of the material. The untreated PBS film obtained via cast extrusion exhibited better barrier properties than polyolefins [33,34] but higher O_2_ permeability than other aliphatic polyesters, e.g., those based on polyhydroxyalkanoates [12] and processed using the same method. HPMC layer improved the barrier properties of the films: 261 and 274 cm^3^/m^2^ × 24 h before and after UV treatment, respectively. It can be seen that the polysaccharide-based layer decreased oxygen permeability; however, after aging, OTR slightly increased. It can be caused by the erosion of the coating during treatment (SEM, Figure 1). The addition of the extract and the ethosomes caused a further decrease in OTR values: 250, 220, 263, and 208 cm^3^/m^2^ × 24 h for ES5, ES5 UV, ET5, and ET5 UV, respectively. Interestingly, the permeability for samples with modified coatings decreased after aging. This phenomenon may be caused by the migration of hydrophobic additives on the surface and the formation of more obstacles for gas particles.

### 3.7. Color Analysis

The effect of coatings and accelerated UV aging on the changes in color, opacity, and transparency of the films is shown in Table 5. The neat PBS films had L*, a*, and b* parameters of 90.15 ± 0.15, −0.13 ± 0.01, and 2.30 ± 0.09, respectively. Despite the intense yellow or orange color of the additives, the presence of modified coatings and film aging had a negligible effect on the color changes in the samples. Although, for the L*, a*, b*, and ΔE parameters, the differences were statistically significant (*p* < 0.05), the ΔE values for almost all films were less than 1 and thus below the color difference detection threshold of the human eye [35]. Only for ET1 the UV value was 1.93 ± 0.04, which can be considered detectable with careful observation. Comparable results for polyester films produced via cast extrusion with HPMC coating with herbal extracts were obtained in the work [13].

The yellowness index ranged from 3.64 ± 0.14 (for the PBS UV sample) to 5.75 ± 0.02 (for ET1 UV). The low values of this parameter, together with the CIELab results, indicate that, despite the use of active additives with strong coloration (orange for the extract and yellow for the ethosomes), the obtained coatings did not show large deviations from neat PBS. This is important from an industrial point of view since the color of the packaging influences consumers’ purchase choices [36].

Despite the lack of human-noticeable differences in the color of the films (for most films, ΔE < 1), accelerated UV aging affected changes in the opacity and transparency of the films. The presence of the additives (ET and ES) in the HPMC layer affected the decrease in transparency of the films subjected to accelerated UV aging. Only for neat PBS the difference in the value of this parameter was not statistically significant (*p* > 0.05).

### 3.8. Antimicrobial Analysis

**Figure 6:**
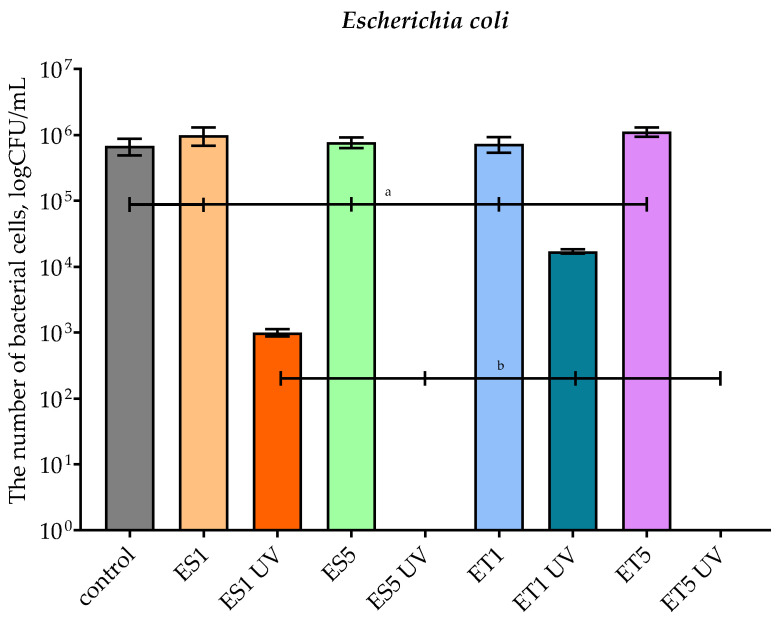
The effect of film samples on the viability of *Escherichia coli* cells. Bars with different lowercase letters are significantly different at *p* < 0.05.

Figure 6 shows the results of testing the antimicrobial properties (against *E. coli*) of HPMC-coated PBS films with scCO_2_ of sea buckthorn extract (ES) or its ethosomes (ET) before and after accelerated aging. The ES1 UV and ET1 UV samples showed a significant reduction in bacterial counts to ~10^3^ and ~10^4^ CFU/mL, respectively, representing a 3-log and 2-log reduction relative to the control. Notably, the samples ES5 UV and ET5 UV showed complete inhibition of the bacterial growth. Samples not subjected to aging showed no antimicrobial activity (counts of 10^6^ CFU/mL). These results indicate that the presence of sea buckthorn bioactive components in combination with UV treatment leads to the effective inhibition of bacterial growth, and that higher additive concentrations (5 pph) after aging are most effective. Combined with the results from the SEM, this suggests a controlled release of active additives from the coating under UV/elevated temperature exposure, which offers the potential possibility of using these films as an active packaging system with antimicrobial properties.

**Figure 7:**
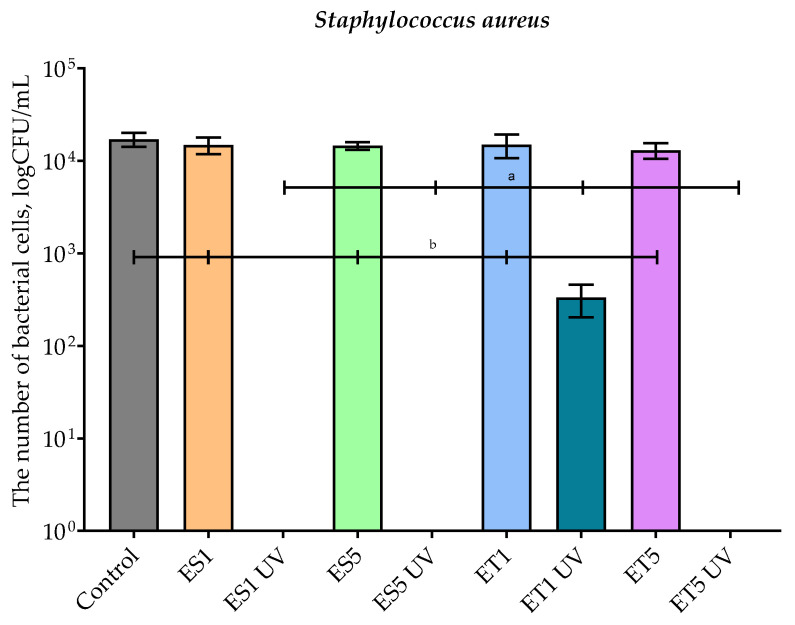
The effect of film samples on the viability of *Staphylococcus aureus* cells. Bars with different lowercase letters are significantly different at *p* < 0.05.

Figure 7 shows the results of the antimicrobial properties of the samples before and after accelerated UV aging against *S. aureus*. As in the case of the test against *E. coli*, the films not subjected to accelerated aging were not effective against Gram-negative bacteria. The bacterial number of viable cells was similar to the number obtained for the control sample. In the case of ES1 UV and ES5 UV, there was a complete absence of microbial growth. Similarly, for the ET5 UV sample, the inhibition of Gram-positive cells was total/complete. Moreover, the irradiated ET1 layer did not cause inhibition reduction in viable microorganisms; however, it reduced their number significantly (close to 2-log), confirming that the concentration of the antimicrobial agent in a coating has an impact on its effectiveness. It has to be added that the ET1 UV coating was less effective than the ES1 UV layer. The reason for the lower activity of the ET1 UV coating could be that the extract as a antimicrobial agent was trapped in ethosomes. Contrary results were demonstrated in a previous study [13], which proved that a polyester film coated with an HPMC layer with *Hypericum* L., *Achillea millefolium* L., and *Hippophae rhamnoides* L. ethanolic extracts inhibited the growth of *S. aureus* and *E. coli.* The reason for the high activity of this coating may be the hydrophilic character of extracts dissolved in the HPMC carrier. Furthermore, accelerated UV aging decreased the activity of the previously mentioned layers, which could have been caused by the release of extract from the coating. In summary, the results confirmed that, after treatment, the active compounds from the extract became exposed but remained within the carrier and, thus, effectively inhibited microbial growth. These outcomes were confirmed by SEM analysis.

**Figure 8:**
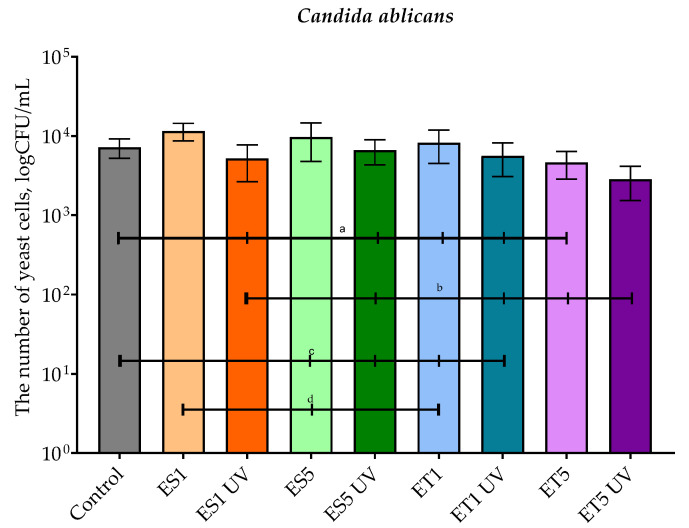
The effect of film samples on the viability of *Candida albicans* cells. Bars with different lowercase letters are significantly different at *p* < 0.05.

Figure 8 shows the results of the tests with *C. albicans* for PBS ES and PBS ET films before and after accelerated UV aging. In contrast with the above-mentioned tests against representatives of Gram-negative bacteria (*E. coli*) and Gram-positive bacteria (*S. aureus*), in the test against a representative of fungi (*C. albicans*), films containing the extract both before and after accelerated aging were not able to inhibit the growth or even significantly reduce/decrease the number of viable yeast cells.

Sea buckthorn extracts have been previously studied for their properties using various parts of the plant (leaves, stem, coleus, and seeds) [37] and solvents [38]. Brobbey at al. demonstrated the antimicrobial activity of ethanolic extracts of sea buckthorn used to modify carboxymethylcellulose as a coating on paper. The study succeeded in obtaining growth inhibition zones for both *S. aureus* (as a representative of Gram-positive bacteria) and *Pseudomonas aeruginosa* (as a representative of Gram-negative bacteria) [39]. Similarly, Feng et al. investigated antimicrobial properties by developing chitosan films modified with sea buckthorn extract. They observed activity against both *S. aureus* and *E. coli*, but the stronger growth inhibition effect was on *S. aureus* [40]. These studies correspond with our results.

For the compositions tested in this publication, no biocidal effect was observed against *C. albicans*, which agrees with literature data. Smida et al. produced pulp sea buckthorn oil-based mouthwash and studied its antimicrobial properties. They were able to find bactericidal activity, but no fungicidal activity was reported [41].

## 4. Conclusions

Given the growing demand for active and biodegradable packaging, there is a need to develop systems that combine functionality, safety, and environmental friendliness. The proposed solution—PBS films obtained by cast extrusion coated with a layer of HPMC containing sea buckthorn extract or its ethosomes forming fully biodegradable material—may represent an innovative approach to food protection, especially stored in conditions with UV exposure and higher temperature (e.g., outdoors). Such a system offers good barrier properties (OTR significantly lower than that of polyolefins and PLA), biodegradability, and antibacterial properties (activated during storage), making it potentially attractive to the food industry. In view of the above factors, the developed systems appear to be a potentially interesting solution in the food industry as packaging that comes into contact with UV radiation, whether naturally emitted by the sun or from artificial sources, for example, during UV activation.

## Figures and Tables

**Table 1:** The acronyms of the films.

Sample	HPMC Coated	Active Additives to HPMC Coating [Parts Per Hundred]	UV Aged
PBS	No	-	No
PBS UV	No	-	Yes
HPMC	Yes	-	No
HPMC UV	Yes	-	Yes
ES1	Yes	1 pph sea buckthorn scCO_2_ extract	No
ES1 UV	Yes		Yes
ES5	Yes	5 pph sea buckthorn scCO_2_ extract	No
ES5 UV	Yes		Yes
ET1	Yes	1 pph sea buckthorn scCO_2_ extract-based ethosomes	No
ET1 UV	Yes		Yes
ET5	Yes	5 pph sea buckthorn scCO_2_ extract-based ethosomes	No
ET5 UV	Yes		Yes

**Table 2:** The water contact angle results.

Sample	Average Contact Angle [°]	Example Images
PBS	63 ± 3	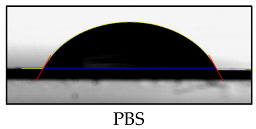
PBS UV	53 ± 6	
HPMC	32 ± 3	
HPMC UV	37 ± 4	
ES1	41 ± 2	
ES1 UV	35 ± 4	
ES5	45 ± 5	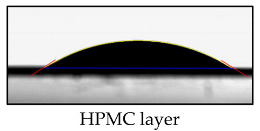
ES5 UV	39 ± 3	
ET1	41 ± 4	
ET1 UV	35 ± 3	
ET5	48 ± 5	
ET5 UV	39 ± 4	

**Table 3:** The number average molecular weight (Mn), the weight average molecular weight (Mw), and the polydispersity index (PDI) of untreated and aged PBS.

Sample	Mn	Mw	PDI
PBS	52,684	115,648	2.20
PBS UV	47,138	110,276	2.34
HPMC UV	45,063	103,793	2.30
ES1 UV	46,472	106,888	2.31
ES5 UV	45,144	105,663	2.34
ET1 UV	47,453	108,535	2.29
ET5 UV	45,188	105,628	2.34

**Table 4:** Thickness and mechanical characteristics (Young’s modulus (YM), tensile strength (TS), elongation at break (EB), maximum puncture force (F_max_), and penetration path (I_s_)) of PBS films.

		Elongation			Puncture	
Sample	Thickness [µm]	YM [MPa]	TS [MPa]	EB [%]	F_max_ [N]	I_s_ [mm]
PBS	82 ± 2 ^a,b,e^	519 ± 70 ^d^	41.9 ± 3.5 ^b,f^	276 ± 31 ^c,d^	3.34 ± 0.11 ^a,b^	0.46 ± 0.10 ^a^
PBS UV	74 ± 4 ^c^	660 ± 62 ^b,c,e^	28.0 ± 7.5 ^d,e^	6.42 ± 2.10 ^a^	3.04 ± 0.12 ^b^	0.43 ± 0.14 ^a^
HPMC	78 ± 2 ^d,f^	636 ± 56 ^a,b,c^	50.1 ± 6.4 ^c^	323 ± 42 ^e,f^	3.21 ± 0.15 ^b,c^	0.59 ± 0.09 ^a,b^
HPMC UV	83 ± 1 ^a,b^	677 ± 575 ^c,e,f^	37.9 ± 3.0 ^a,b^	21.2 ± 8.5 ^a,b^	3.15 ± 0.26 ^a,b^	0.47 ± 0.10 ^a^
ES1	80 ± 1 ^e,f^	579 ± 48 ^a,d^	48.3 ± 4.3 ^c^	327 ± 14 ^f^	3.98 ± 0.38 ^d^	0.56 ± 0.20 ^a,b^
ES1 UV	76 ± 2 ^c,d^	612 ± 52 ^a,b,c^	27.2 ± 4.3 ^d^	7.21 ± 2.27 ^a^	3.97 ± 0.28 ^d^	0.53 ± 0.19 ^a^
ES5	84 ± 3 ^a,b^	634 ± 37.7 ^a,b,c^	47.7 ± 3.0 ^c,f^	297 ± 14 ^c,e^	3.34 ± 0.09 ^a,b,c^	0.44 ± 0.09 ^a^
ES5 UV	82 ± 1 ^a,e^	606 ± 48 ^a,b,c^	33.6 ± 3.8 ^a,e^	33.9 ± 4.5 ^b^	3.95 ± 0.14 ^d^	0.53 ± 0.05 ^a^
ET1	76 ± 4 ^c,d^	745 ± 55 ^f^	38.3 ± 4.9 ^a,b^	252 ± 17 ^d^	3.56 ± 0.25 ^a^	0.57 ± 0.09 ^a,b^
ET1 UV	75 ± 3 ^c^	720 ± 93 ^e,f^	36.9 ± 4.7 ^a,b^	13.7 ± 1.1 ^a,b^	3.32 ± 0.28 ^a,b,c^	0.48 ± 0.23 ^a^
ET5	84 ± 2 ^a,b^	588 ± 55 ^a,b,d^	38.2 ± 3.2 ^a,b^	292 ± 17 ^c^	3.57 ± 0.18 ^a^	0.41 ± 0.12 ^a^
ET5 UV	85 ± 3 ^b^	640. ± 62 ^a,b,c^	34.6 ± 3.6 ^a^	71.2 ± 14.5 ^g^	3.89 ± 0.39 ^d^	0.75 ± 0.31 ^b^

Values are presented as means ± standard deviations. Means with different lowercases are significantly different at *p* < 0.05.

**Table 5:** Color (L*, a*, and b*), total color difference (∆*E*), yellowness index (*YI*), opacity, and transparency (transmittance at 700 nm) of unmodified and modified PBS films.

Sample	L*	a*	b*	ΔE	YI	Opacity	T [700 nm]
PBS	89.52 ± 0.10 ^g^	−0.16 ± 0.15 ^c^	2.82 ± 0.08 ^a,b^	Used as standard	4.46 ± 0.12 ^a^	12.6 ± 0.3 ^a^	58.5 ± 1.1 ^e^
PBS UV	90.15 ± 0.15 ^i^	−0.13 ± 0.01 ^c,d^	2.30 ± 0.09 ^c^	0.83 ± 0.17 ^i^	3.64 ± 0.14 ^c^	11.6 ± 0.2 ^f^	58.7 ± 0.4 ^e^
HPMC	89.95 ± 0.09 ^a,c^	−0.54 ± 0.01 ^e^	2.98 ± 0.05 ^e^	0.68 ± 0.06 ^a,b^	4.73 ± 0.09 ^e^	11.1 ± 0.3 ^e^	54.6 ± 0.4 ^c,d^
HPMC UV	90.01 ± 0.07 ^c,d^	−0.43 ± 0.01 ^a,b^	2.83 ± 0.05 ^a^	0.63 ± 0.04 ^b,c^	4.50 ± 0.08 ^a,b^	13.2 ± 0.7 ^c^	52.4 ± 0.6 ^b^
ES1	90.07 ± 0.10 ^b^	−0.40 ± 0.01 ^f^	2.69 ± 0.05 ^b^	0.67 ± 0.10 ^c^	4.26 ± 0.08 ^d^	13.6 ± 0.2 ^d^	55.8 ± 0.7 ^a,d^
ES1 UV	89.91 ± 0.06 ^e^	−0.41 ± 0.00 ^c,d^	2.85 ± 0.03 ^i^	0.53 ± 0.04 ^a^	4.54 ± 0.05 ^b^	13.7 ± 0.2 ^d^	46.4 ± 0.9 ^f^
ES5	89.65 ± 0.05 ^h^	−0.55 ± 0.00 ^f^	3.41 ± 0.03 ^a^	0.79 ± 0.02 ^g^	5.44 ± 0.05 ^h^	12.3 ± 0.2 ^b^	53.9 ± 0.5 ^c^
ES5 UV	89.91 ± 0.07 ^f^	−0.45 ± 0.01 ^d^	3.06 ± 0.05 ^g^	0.62 ± 0.01 ^a^	4.86 ± 0.08 ^f^	12.9 ± 0.3 ^a^	47.8 ± 0.0 ^g^
ET1	89.60 ± 0.02 ^d^	−0.34 ± 0.00 ^b^	2.79 ± 0.01 ^d^	0.28 ± 0.01 ^f^	4.45 ± 0.02 ^a^	12.3 ± 0.2 ^b^	56.6 ± 0.8 ^a^
ET1 UV	87.70 ± 0.04 ^a^	−0.14 ± 0.01 ^a,b^	3.53 ± 0.01 ^a^	1.93 ± 0.04 ^d^	5.75 ± 0.02 ^i^	12.8 ± 0.2 ^a^	51.8 ± 0.6 ^b^
ET5	89.74 ± 0.05 ^b^	−0.33 ± 0.01 ^e^	2.86 ± 0.02 ^h^	0.36 ± 0.02 ^h^	4.55 ± 0.04 ^b^	12.7 ± 0.2 ^a^	56.8 ± 0.3 ^a^
ET5 UV	88.96 ± 0.03 ^a^	−0.10 ± 0.02 ^a^	3.30 ± 0.03 ^f^	0.73 ± 0.04 ^e^	5.30 ± 0.06 ^g^	13.2 ± 0.3 ^c^	50.3 ± 0.4 ^h^

Values are presented as mean ± standard deviation. Means with different lowercases are significantly different at *p* < 0.05.

## Data Availability

The original contributions presented in this study are included in the article. Further inquiries can be directed to the corresponding author.
